# A ten-year search for synchronous cells: obstacles, solutions, and practical applications

**DOI:** 10.3389/fmicb.2015.00238

**Published:** 2015-03-25

**Authors:** Charles E. Helmstetter

**Affiliations:** Department of Biological Sciences, Florida Institute of TechnologyMelbourne, FL, USA

**Keywords:** synchronous cells, cell cycle, cell division, chromosome replication, *E. coli*, cellular baby machine

## Abstract

My effort to use synchronously dividing cultures to examine the *Escherichia coli* cell cycle involved a 10-year struggle with failure after failure punctuated by a few gratifying successes, especially at the end. In this essay, I recount my personal journey in this obsessive experimental pursuit. That narrative is followed by a description of a simplified version of the “baby machine,” a technique that was developed to obtain minimally disturbed, synchronously growing *E. coli* cells. Subsequent studies with this methodology led to an understanding of the basic properties of the relationship between chromosome replication and cell division. Accordingly, I end this reminiscence with a simple, fool-proof graphical strategy for deducing the pattern of chromosome replication during the division cycle of cells growing at any rate.

## Introduction

Frank Sinatra once famously sang about age thirty-five being a very good year. For me, that happened in 1968. It marked the end of a long, exhausting, but ultimately satisfying expedition to decipher the growth-rate-dependent coordination between chromosome replication and cell division in *E. coli.* Here I describe the backstory to the development of the “baby machine” technique that ultimately led to our description of the *E. coli* cell cycle published in 1968 (Cooper and Helmstetter, 1968; Helmstetter and Cooper, 1968; Helmstetter et al., 1968). At the conclusion of this personal account, I attempt to dispel any impression that there are major complexities associated with either performing the baby machine procedure, or perhaps of more pertinence, with deciphering the growth-rate dependency of chromosomal replication patterns. Both undertakings are actually quite simple to carry out, as I endeavor to explain with a few relatively painless illustrations.

## A research plan

My interest in the bacterial division cycle began in 1958 while a graduate student with Robert B. Uretz in the Committee on Biophysics at the University of Chicago. It was an unexpected shift in direction because I had previously become fascinated with atomic and radiation physics as a physics major at Johns Hopkins University. My penchant for physics continued and expanded while in masters programs in biophysics at the University of Michigan and in radiological physics at the University of Chicago. As a consequence, I entered the Ph.D. program at Chicago, and joined Bob's laboratory, with the intent of becoming a radiation biologist. The shift in career plans came about after reading some of the stunning work on bacterial conjugation produced at the Pasteur Institute in the 1950s (e. g., Wollman and Jacob, 1955; Wollman et al., 1956). I had no idea scientific research could be so exciting. So after reading everything I could find on the topic, it was clear that I had to study some aspect of *E. coli* DNA.

The decision to focus on chromosomal DNA in the cell cycle came about naturally because Bob Uretz and the Committee on Biophysics at Chicago were renowned for microbeam irradiation of mitotic chromosomes (Uretz et al., 1954), and I had already spent many hours observing and filming the response of newt heart cell chromosomes to UV microbeams. Furthermore, the surprising paucity of information available on the cell cycle of bacteria, compared to that of eukaryotes, was obvious and intriguing. Plus, I could then combine my new interest in bacterial genetics with the laboratory's interest in radiobiology by investigating cell cycle-dependent sensitivity to photo-inactivation by DNA intercalating agents. It seemed quite straightforward at the time. To initiate my career in cell cycle research, all I needed to do was synchronize *E. coli* and I was off. How naïve.

## Synchronous cells

My search for synchronously dividing *E. coli* began in late 1958. Unfortunately, it took 4 years to come up with an acceptable technique, another 4 years to finally figure out an optimal way to apply it, and two additional years to generate and correctly interpret key data on the division cycle. What an unexpectedly long and difficult experience that was. Of course there were personal milestones along the way, including the births of sons Charlie and Michael, but without sheer stubbornness in the face of endless setbacks, my research career would have collapsed on more than one occasion.

The first step was to choose a strain of *E. coli.* I don't recall how I came to the decision to choose *E. coli* B/r, but it was likely because Aaron Novick had been running a bank of chemostats containing strain B in an adjacent laboratory (Novick and Szilard, 1950). I was also aware of Evelyn Witkin's radiation resistant mutant B/r (Witkin, 1946) and felt comparing a radiation resistant mutant with the parental strain might be useful. Also, strain B/r appeared to form fewer filaments during exponential growth, a seemingly advantageous property. That decision turned out to be the first of two incredible strokes of pure good luck during this investigation. If the far more popular strain K-12 had been chosen, most of the crucial experiments I will mention later would likely have failed and the baby machine technique would not have been developed, at least by me^1^.

In the late 1950s there were basically three different approaches reported for bacterial synchronization: single or multiple temperature shifts, single or multiple nutritional deprivations, and size selection by filtration or centrifugation (Helmstetter, 1969). I tried all of them over and over with limited success. These were not trivial efforts because cell concentrations were all determined using agar plates, requiring pouring, plating, counting, and cleaning hundreds of glass Petri dishes every week. That was the case until 1 day a guy named Joe Coulter, who along with his brother had started a small electronics firm in Chicago, walked into the laboratory carrying a machine he claimed could accurately count thousands of bacteria in seconds. After he had considerable difficulty getting mercury to fill the new manometer during set-up of the instrument (not uncommon for an inexperienced user but a bit surprising in retrospect for someone named Coulter), it worked! This was, of course, the original Coulter Counter model A. Now I was able to not only get rapid, accurate cell concentrations, but I was also able to simultaneously see the “sizes” of the cells on the integrated oscilloscope. With the model A amplifiers and a 30-μ m aperture tube, the size distributions of exponential-phase, newborn and synchronously growing cells were readily distinguishable. This instrument proved to be invaluable for my work, and the work of many others who dared try to employ synchronously dividing cells.

Since the goal in studies of this sort was to examine the cell cycle properties of cells in exponentially growing cultures undergoing unrestricted growth, the technique for producing the synchrony must cause little if any disturbance to growth. At a minimum, it was required that the cells undergo at least two cycles of detectable synchrony, that whatever is observed in the first cycle repeats in the second, and that the fundamental properties of the synchronous cultures, such as cell sizes and growth rates, mimic the initial exponential-phase populations. All methods I tested caused some level of disturbance. Many methods produced a single burst of seemingly synchronized division after a delay period, but these were deemed to be nothing more than a reflection of the recovery from the sometimes harsh treatments employed. The method that appeared to cause the least disturbance involved filtration of a culture through a stack of Whatman cellulose filter papers. In this procedure, the smaller newborn cells pass through the stack into the effluent, and can be collected, while the larger, older cells are retained within the stack (Maruyama and Yanagita, 1956; Abbo and Pardee, 1960). With some modifications from the original reports, such as pressure rather than vacuum filtration, this technique enabled me to perform a few simple irradiation experiments on cells at very low concentrations.

During this time, two fellow graduate students with whom I had close contact, and who would eventually play important roles in this work, were Donald J. Cummings and David Friefelder. Don was working on T2 bacteriophage structure with Lloyd Kozloff, and Dave was a member of the Uretz lab. Don and I became very close friends and spent many hours gabbing about our work, often during endless games of bridge in smoke-filled living rooms. This friendship with Don proved invaluable after graduation. Don received his degree before I, did a postdoc with Ole Maaløe at the University Institute of Microbiology in Copenhagen, and then accepted a position at the National Institutes of Health in Bethesda to continue his work with T2 in a unit headed by Ernst Freese. I, on the other hand, was committed to 2 years of active duty service after being enrolled in the Army Reserved Officers' Training Corps at Johns Hopkins. Fortunately I was able to transfer from the Army to the Commissioned Corps of the U. S. Public Health Service, and to serve my time at NIH, thanks to a convincing letter to whomever makes these transfer decisions and the blessing of Freese. So in the fall of 1961, I was given a desk in Don's laboratory, as well as a small room down the hall with a walk-in incubator and an indispensable model A Coulter Counter in order to continue developing the filtration synchrony technique.

## Origin of the baby machine

At NIH my plan was to increase the quantity of small cells produced with the filtration technique, while minimizing perturbations, by employing a larger vessel capable of rapidly pushing liters of cells through a thick stack of filter papers. To accomplish this, a skilled machinist constructed a filter holder from a large stainless steel pipe, 12 cm in diameter and 40 cm long. A removable cap was clamped to the top and attached to a cylinder of compressed air to produce the pressure needed to push the culture through the filter stack. When it was completed, Don and I walked across campus to the machine shop to run the first test. After it was all set up and the top was clamped securely and attached to the air cylinder, we all stood as far back as we could and one of us, the machinist I believe, turned the valve on the cylinder. There was an immediate sound of a canon having been fired in this cavernous, high-ceilinged room. At the time I thought the entire apparatus had been blown into the air with a thunderous roar. We all dove for cover. It turned out to be just the cap. The only subsequent sound was my voice saying “has it come down yet?” a comment Don joked about for many years thereafter with anyone willing to listen to this story about our underestimation of the importance of pressure regulation. The device was eventually properly strengthened and regulated, and I began running tests with it.

Everything changed in early 1963 while at a meeting of the Biophysical Society in New York. After one of the sessions I was standing at the back of a room with a small group talking about synchronizing cells. I believe Philip Hanawalt was amongst us, and I certainly recall David Friefelder being there too because he asked me a question that completely changed the course of the work. Dave loved back-and-forth banter, perhaps originating early in life while he was one of the teenage stars of a very popular radio show in the U. S. called Quiz Kids. After describing the technique I was using, Dave asked a number of questions including how long the filtration took. I said, “a few minutes.” He then said, as I distinctly recall, “well then, the cells must be growing in the filter stack.” Although I felt at the time that Dave's comment was intended to be a criticism of the method, it turned out to be the second incredible stroke of good luck during this pursuit of synchrony.

The story of what happened later that night has been told previously (Cooper, 1997), but I will recount it from my perspective. I couldn't sleep as I mulled over Dave's comment while staring at the ceiling in the dark hotel room. I was thinking about the larger cells stuck in the filter stack growing and dividing while the medium passed through for those short few minutes. Then for reasons unknown, since I know nothing about poultry farming, I began visualizing chickens attached all over the ceiling of the room and thinking that the only (living) objects that would fall from the surface would be eggs. That was it. I thought that if cells became stuck within the filter stack while culture medium passed through, the only cells that could be released from the stack would be newborn cells originating from the portion of the adhered cells that was not involved in their adherence I realized that many of the dividing rod-shaped cells might not release progeny at all, or that some newborn cells might reattach, but in the ideal case in which attachment was permanent, only new daughter cells would be released, no matter how few. Experimentally, strong attachment and good flushing with culture medium ought to yield a highly pure population of newborn cells. The best part was that this could be a truly minimally disturbed synchronous population because the process of preparing the synchronous cells simply involved collecting cells falling from a surface-bound culture growing under undisturbed conditions.

I was feeling euphoric during the train ride back to Bethesda and I very likely went to the lab that night to set up a culture to test the idea the next day. I don't recall the precise filter configuration for the first test, but in essence I performed the usual filtration of cells through the filter stack. However, instead of collecting the first small cells released I simply kept fresh medium flushing slowly through the filter containing whatever cells remained bound. During the first few minutes of the experiment, the concentration of cells in the effluent decreased to almost background. (At that time, it had not yet occurred to me that the cells close to division might be so completely attached that neither daughter would release at division). Then, to my relief, the concentration began to increase gradually. I could see in the oscilloscope of the Coulter Counter that most of the cells were at the size expected for newborn cells. Soon the cell number rose rapidly, reaching a peak at about 40 min with a thick pattern of spikes in the oscilloscope, seemingly all at newborn size. What a moment. I had to stop the experiment earlier than I would have liked due to an appointment for a haircut in a shop in the basement of the NIH Clinical Center. So the first person I told about this successful experiment was a very disinterested barber.

**Figure 1:**
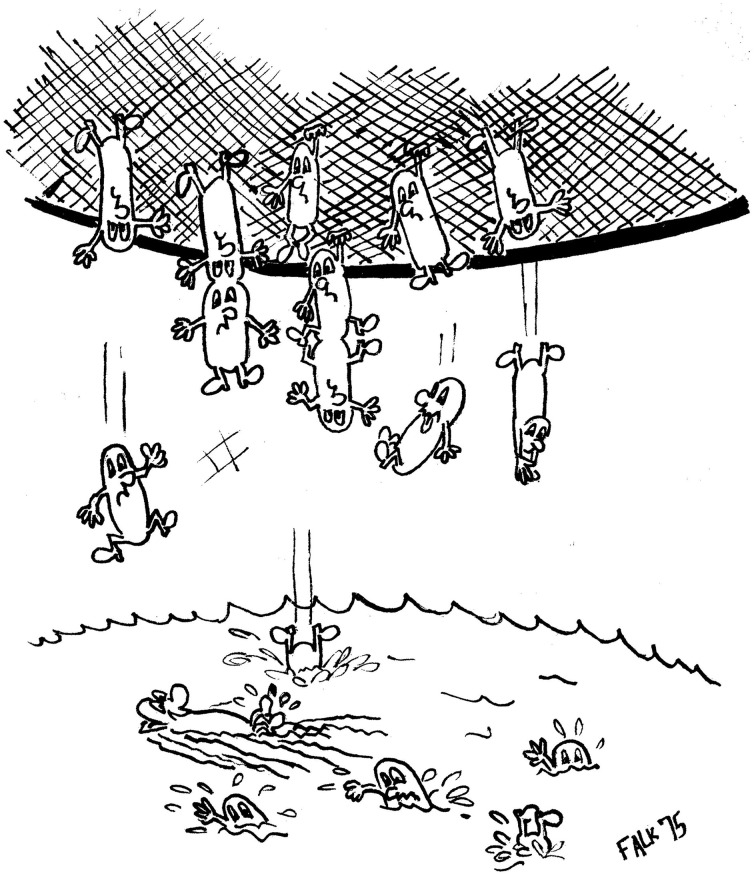
**Bacterial baby machine cartoon**. Caricature of the technique sketched by Avshalom Falk while a student in the laboratory of Eliora Ron at Tel Aviv University.

Development and testing of this new approach progressed rapidly, with the final configuration consisting of simply filtering the cells onto a nitrocellulose membrane filter, inverting the filter apparatus, pumping medium through the filter, and collecting the cells that fell off (Helmstetter and Cummings, 1964). It eventually became known as the “baby machine,” and depicted with humor (Figure 1). I don't recall who first called it by that name, but it wasn't me since I continued to dub it “membrane elution,” true to my conservative writing style.

## A key experiment in copenhagen

With the method a success and my tour with the Public Health Service ending in the fall 1963, I headed to Ole Maaløe's laboratory in Copenhagen on a 1-year NIH postdoctoral fellowship. This was a big deal for me since I was well aware that Ole's lab was at the top of my new field of microbial physiology, and Don Cummings had raved about his experiences there. I also assumed, correctly, that Maaløe would have keen interest in having this new minimally disturbing synchrony technique introduced into his lab. It was a scientifically stimulating year with an eclectic group of North Americans: James Friesen, Steven Cooper, D. Joseph Clark and Kivie Moldave, along with Danish colleagues Niels Ole Kjeldgaard and Knuth Rasmussen. I was able to absorb much of the beautiful ground-breaking work on bacterial physiology that had come out of the laboratory (e.g., Schaechter et al., 1958; Hanawalt et al., 1961). The Copenhagen School motto of “look but don't touch” when investigating microbial physiology became embedded for life. I also developed a multi-year fascination with possible explanations for the “rate maintenance” phenomenon, that is, the finding that after a shift of exponentially growing *Salmonella typhymurium* to a richer culture medium, the rate of division remained essentially unchanged for 60–70 min at 37°C before shifting suddenly to the rate expected in the new medium (Kjeldgaard et al., 1958).

Shortly after arriving, I set up the baby machine apparatus in an incubator room that contained a small opening hatch in one wall of the laboratory Joe Clark and I occupied, thereby enabling me to collect samples in comfort. For health reasons I accomplished very little, and did not interact with Maaløe as much as anticipated, except for some discussions on drafts of the monograph on macromolecular synthesis he and Kjeldgaard were writing (Maaløe and Kjeldgaard, 1966). However, he was enormously kind and helpful to me when I became ill, ferrying me around town to find the best physicians, even to the extent of going into examining rooms with me to “participate” in the physical exams.

But one experiment was performed that would eventually prove to be very important on the way to deciphering the replication-division coordination in *E. coli.* We all spent a good deal of time in scientific bull-sessions discussing what we thought we knew about the cell cycle. One day Steve Cooper and I decided to do an unusual experiment with the baby machine. We decided to pulse-label the cells with ^14^C-thymidine immediately prior to binding them to the membrane filter, rather than labeling the synchronous cells eluted from the surface in the usual manner, with the idea that we could learn something about chromosome segregation. So we pulse-labeled glucose-grown *E. coli* B/r, collected newborn cells eluted from the membrane for about three generations, and measured radioactivity per cell. The results were very clear. The radioactivity per cell decreased in a step-wise pattern, with horizontal plateaus followed by sudden two-fold decreases every generation. As I remember, we concluded that the two-fold reduction in radioactivity per cell each generation was the relatively uninteresting consequence of semiconservative replication followed by random distribution of chromosomal DNA into daughter cells, and that we had learned little of real value. What a mistake. The mistake was probably a consequence of our inability at that time to accurately measure, and thus interpret, the characteristic fluctuation in concentration of newborn cells eluted from a surface-bound population. Unfortunately it would take me two more years to think about this experiment again and interpret it correctly.

## Dealing with adversity

In the fall of 1964, when it became time to find a job as my year in Copenhagen neared an end, I was pleasantly surprised to find that the mid 1960s was a time when positions in science were plentiful. Due to my obsession with using the baby machine to study basic properties of chromosome replication in *E. coli*, I preferred a full-time research position rather than a university appointment. One day I got a letter from someone at Roswell Park Cancer Institute describing a research position that included the option of joining the graduate faculty at what would become the State University of New York at Buffalo. I had not heard of the place so I looked on a map of Buffalo for a park named Roswell, without success. (I soon learned that in 1898 Dr. Roswell Park founded the nation's first research facility devoted exclusively to cancer research at the University of Buffalo). With some trepidation I accepted an offer to fly to Buffalo, see the facilities, and meet faculty. After being convinced I could focus on the research of my choosing while an active member of the graduate program, and after meeting several first-rate researchers such as Kenneth Paigen, David Pressman, Theodore Hauschka and David Harker, I accepted the position. It didn't hurt that I was offered a salary that was about 50% higher than I had anticipated.

The NIH grant application I submitted while in Copenhagen was funded before I arrived in Buffalo, and that, along with some small start-up funding, got me going. The next 2 years were enjoyable personally but painful scientifically. My students, technicians and I must have performed several hundred experiments with synchronous cells obtained with the baby machine. Some worked quite nicely but the ones I really cared about were never as pristine as required. The questionable experiments all involved pulse-labeling synchronous cultures with radioactive precursors of various macromolecules, with primary interest in nucleic acids. I was distressed by what appeared to be minor but persistent inconsistencies in the uptake patterns in the first and second cycles of synchronous growth. Nothing I tried solved the problem. I vividly remember the very cold Buffalo night when I finally came to the decision, while leaving the lab and heading to the basically deserted car park to scrape the ice from my windshield, that I, at least, was incapable of growing *E. coli* synchronously without noticeable disturbance. What a miserable night. I simply could not understand why a technique that seemed so sound theoretically was not working properly^2^. That night I became firmly convinced that I had better do something else with my life because 8 years of beating my head against the wall was enough.

Of course that is not what happened. I spent the next few days mulling over the concept behind the baby machine and what fundamental flaw I might be missing. For whatever reason, the experiment Cooper and I did in Copenhagen crossed my mind, and something just snapped. I finally realized what the experimental result was telling us; namely, the rate of ^14^C-thymidine incorporation during the division cycle. It can be understood by considering the following simple facts with reference to that experiment. The first newborn cells to fall from the surface at the start of elution were progeny of the oldest cells in the culture at the time of pulse-labeling with ^14^C-thymidine. The cells eluted at the end of the first generation of growth on the surface were progeny of the youngest cells in the culture at the time of labeling. Thus, the radioactivity in the newborn cells eluted during the first generation reflected the rate of thymidine incorporation during the division cycle of their parents, in reverse. Similar analysis applied to subsequent generations of elution (see Helmstetter, 1967 for a detailed illustration of this reasoning). What a feeling it was to suddenly realize how powerful this application of the technique would be to study a host of cycle-dependent phenomena with virtually no disturbance of the cells. As with the chicken epiphany, I was fully convinced it would work because all treatments, including labeling, would be done in untouched cultures. All we were asking was for the cells to divide on the membrane surface in the same sequence as they would have divided in the original exponentially growing culture. The first experiment was done the next day. I pulse-labeled a culture with radioactive thymidine, filtered it onto the membrane in the usual way, and I then had to head off to teach a class while a technician collected the samples and prepared them for counting. That experiment failed because I had added too much label, but it was repeated the next day with great success showing unequivocally that in glucose-grown cells, the rate of DNA replication appeared constant for the first half of the cycle, doubled at mid-cycle, and was constant at twice the rate for the second half of the cycle.

## Deciphering the coordination between chromosome replication and cell division

This time my barber wasn't the first to be told about an experimental success. I immediately wrote Steve Cooper a very long letter explaining the benefit and potential of this application of the technique along with numerous sketches of the data. Steve responded with 43 pages of typical enthusiastic verbal eurekas that literally jumped off the pages with ideas for many year's worth of experiments, presumably for me to do. It turned out, however, that by pure chance, Steve, who was in Kivie Moldave's laboratory at Tufts University at that time, had accepted a position with Robert Guthry at Childrens Hospital in Buffalo. Upon arriving in Buffalo in the summer of 1966 his facilities were not yet ready at Childrens so he came to my lab at Roswell with the idea that we could work together for a month or two, since cell cycle research had continued to be one of his interests. By that time I had completed work on chromosome replication in slower-growing *E. coli* B/r, finding a G2-like gap in DNA synthesis (Helmstetter, 1967), and the obvious next step was to extend the analyses to rapid growth in an effort to generalize the findings.

**Figure 2:**
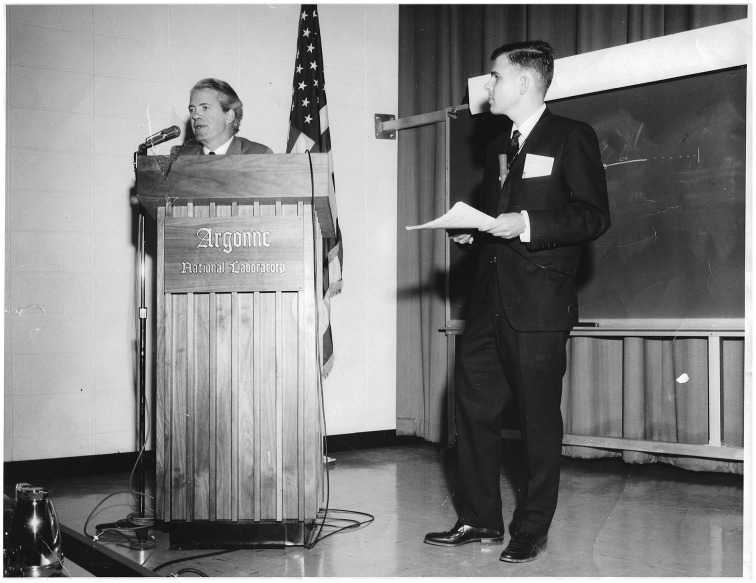
**Argonne National Laboratory, 1968**. Ole Maaløe facilitating discussion after a presentation by Charles Helmstetter at a Division of Biological and Medical Sciences symposium.

After a few ups and downs, humorously described in Cooper (1997), the relationship between chromosome replication and cell division became obvious. Two papers were then written and published in 1968 (Cooper and Helmstetter, 1968; Helmstetter and Cooper, 1968). The first contained new data on ^3^H-thymidine incorporation during the cycle of rapidly growing cells, and the second described a “model” to explain the general relationship between replication and division. The writing was a joint effort although I took primary responsibility for the first and Steve for the second. That division of labor proved wise due to Steve's skill at inventive prose. The first paper reflects my traditional, generally accepted writing style, segmented into: Introduction, Methods, Results, and Discussion. Steve simply wrote in a manner he felt was most informative, irrespective of expected norms. I have to believe his compositional skills are one reason the basic ideas in the second paper came across so easily to readers and are so frequently cited. Another reason lies in the presentation of the findings as a “model” when in fact it was primarily a description of our data and not a generalized model. The “model” states that the time for a round of chromosome replication (*C*) and the time between the end of a round of replication and cell division (*D*) are constants over a specific range of growth rates. Thus, cell division takes place *C* + D min after initiation of each round of chromosome replication. Subsequently, the model was extended to include (*I*), defined as the interinitiation time, i.e., the time required for the cell to achieve the potential to initiate chromosome replication (Helmstetter et al., 1968). Accordingly, in a purely phenomenological sense, *E. coli* duplication can be described as *I* + C + D, irrespective of the durations of *I*, *C*, and *D*. Later that same year, Ole Maaløe and I reconnected at Argonne National Laboratory during a presentation of the model (Figure 2).

I was not entirely comfortable with emphasizing that *C* and especially *D* were constants at growth rates between 1 and 3 doublings/h at 37°C (although that turned out to be a reasonable and useful concept), but I was convinced of the “*ICD*” idea. I was also gratified that the model explained the previously mentioned “rate maintenance” phenomenon, since it stipulated that the rate of cell division would be expected to remain unchanged for *C* + D min after a shift-up (Helmstetter et al., 1968). Plus, being a novice physicist, I saw the possibility of using this simple idea to develop quantitative expressions for the chromosomal DNA contents of cells in exponentially growing cultures. I was not particularly skilled in calculus but I loved the challenge. So while simultaneously listening to the Indianapolis 500 auto race in May 1967, I set out to calculate the genome equivalents of DNA per cell as a function of growth rate in log-phase cultures. It was great fun for someone with modest ability in math, and if you glance at the equations shown in Cooper and Helmstetter (1968), the derivations might seem fairly simple, but it wasn't easy for me. As I thought about the preliminary equations that do not appear in the paper, I found the need to simultaneously envision expressions for the three distinct DNA replication intervals present in the more complex division cycles. Exasperatingly, one or two kept slipping away until at last I was able to retain all three at once, saw the formula, and quickly wrote it down. From there on, and in concert with Steve and Olga Pierucci, the equations flowed out and it was done. I suppose the process of holding multiple mathematical expressions in one's head when deriving equations is common for those with expertise in this area, but it was new to me. I tell this anecdote because I enjoyed doing these and subsequent calculations using the model, but also because it echoes a story I read a while ago about Isaac Newton during the time he was developing calculus. The story I recall is that the staggering genius of Newton was reflected in his apparent ability to hold several mathematical expressions in his brain simultaneously for days at a time, while walking around in London or on his mother's farm, as he meshed them together until the problem of interest was solved. Having been able to retain only three expressions for probably less than a second with considerable effort, I was amazed by this account of an incomprehensible talent of an unquestioned genius.

**Figure 3:**
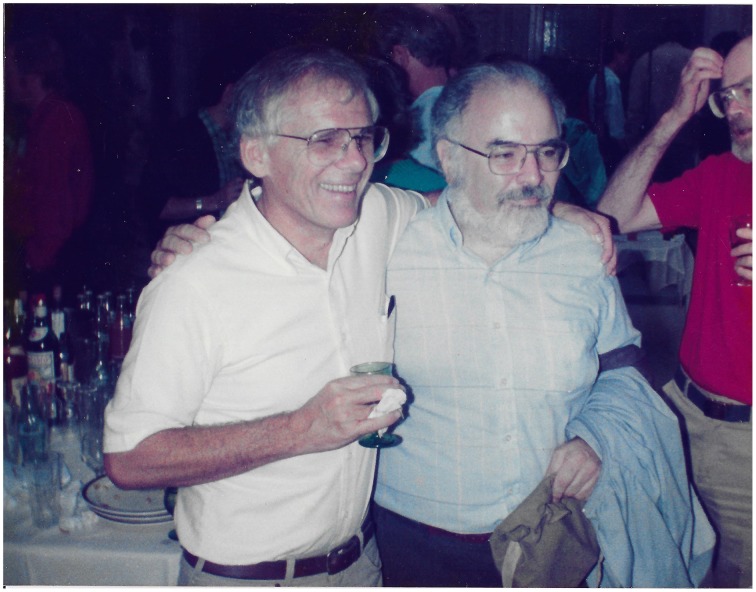
**Charles Helmstetter and Steve Cooper during a reception at a conference in 1987**.

The preceding was intended to describe some of the activities that went into our early contributions to research on specific aspects of the bacterial division cycle. It is not comprehensive and it only reflects our work and not those of numerous others whose studies during the same time frame all contributed to the accomplishments in this field in the late 1960s. In particular, the design of our experiments was made possible by the critical discoveries of Schaechter et al. (1958) on the relationship between cellular properties and culture conditions. Additionally, interpretation of our findings in rapidly growing cells was facilitated by the earlier report of multifork DNA replication in *Bacillus subtilis* (Yoshikawa et al., 1964). I will never know whether the work described here would have been performed, or interpreted correctly, absent these prior findings. Steve Cooper left Buffalo in 1970 for the University of Michigan, but we remained in touch for years, including at the occasional meeting (Figure 3). Several colleagues whose published work, private discussions and encouragement were of great help during this time have already been mentioned, but I also wish to especially acknowledge my long-term collaboration with Olga Pierucci, a number of valuable inputs from K. Gordon Lark, Arthur Pardee and Joe Clark, and the important insightful subsequent contributions of Alan C. Leonard.

## A simplified baby machine

My primary reasons for agreeing to write this exhumation of the past are contained in this and the following section. So if you have skimmed through the preceding, but have a developing interest in synchronous cells or the bacterial cell cycle, this is the point at which you might consider reading more slowly. I completely understand why someone might be reluctant to embark on studies with minimally disturbed synchronous cells if it is believed that the baby machine device is too complex to construct and use. In my biased opinion, this notion is inaccurate. The baby machine process for generating synchronous cells is remarkably simple to perform.

What is actually needed to get minimally disturbed synchronous cells? Nothing very special. In fact, if you would like to try the technique, or perform a few experiments, or test some cells, it is very easy to construct and operate a minimalistic but functional baby machine. The primary requirements are the following:

1. A 150-mm ID porcelain Buchner funnel.
2. A 142-mm diameter, 0.22-μm Millipore MF membrane filter, or equivalent.
3. A mesh screen to place underneath the membrane filter.
4. A means to hold the membrane filter in place in the funnel.

**Figure 4:**
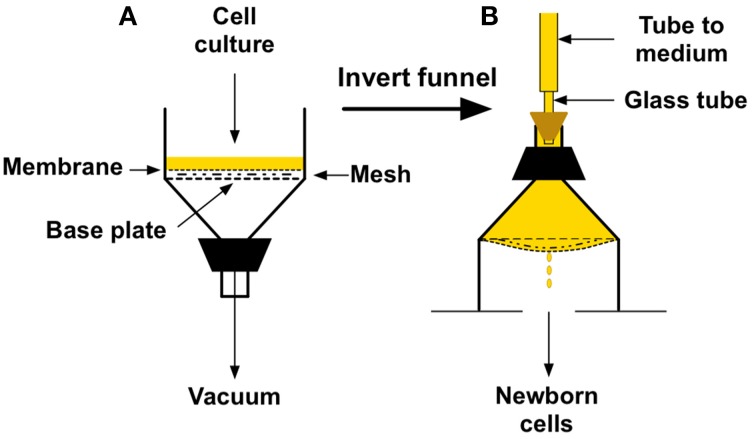
**Buchner funnel version of a cellular baby machine. (A)** Funnel set-up for binding cells to a membrane filter surface. **(B)** Orientation and appearance of the funnel during elution of newborn cells from the membrane filter surface.

Figure 4 shows the basic set-up and procedure using a Buchner funnel with a 150-mm diameter perforated base plate (Scientific Equipment of Houston). To prepare the funnel as shown in Figure 4A, the membrane filter must be sealed to the bottom plate of the funnel. This is most easily accomplished by first running a narrow bead of biologically safe silicone sealant (such as Factor II A-4100 or aquarium-safe silicone) around the bottom of the funnel just inside the 142-mm circumference of a membrane filter. Next, place the mesh screen loosely inside the bead. I recommend using a 120-mm diameter screen cut from a Buchner polyethylene disc (Avogadro's Lab Supply, Inc.). Lastly, place the membrane filter on top of the bead and press down on the edge. I have used the top portion of 130-mm two-piece polypropylene Buchner funnel for this purpose (Avogadro's Lab Supply, Inc.), but any ring of that approximate diameter should work well. After curing, this procedure yields a perfect seal, produces a surface of about 120 mm in diameter for attachment of the cells, and enables culture medium to flow uniformly across the membrane after inversion of the apparatus. The seal is easily removed with a razor blade for the next experiment.

To perform the procedure, a total of 0.5 to 1.0 × 10^10^ bacteria growing in 100–200 ml of minimal defined medium are filtered slowly (1–2 min) under vacuum onto the membrane filter at the appropriate temperature in a warm room or any convenient table-top incubator (Figure 4A). The entire filter holder is then inverted (Figure 4B), culture medium is poured into the top of the inverted holder (about 300 ml), and tubing from a reservoir of medium is connected to the stem of the funnel with a stopper. To produce a sealed system, the tubing should be attached to a narrow glass or rigid plastic tube installed in a hole bored through a stopper of appropriate size, as shown. It is helpful to have most of the stem of the funnel cut off, as indicated in the figure, to ease pouring of the medium and connection of the stopper. A peristaltic pump can be used to regulate the rate of medium flow into the apparatus after inversion. It should be set at about 15 ml/min for a few minutes to flush off weakly attached cells and then reduced to 2 ml/min thereafter. Alternatively, if you wish to perform tests before obtaining a pump, a reservoir bottle with a bottom outlet can be placed above the apparatus with a hose clamp acting as the flow regulator. In either case, once medium flow begins, the underside of the membrane will become convex with drops coming from the center as seen in Figure 4B. The smooth, convex shape of the membrane is VERY important because it enables the medium to flow uniformly over the surface of the attached cells, and to form drops that fall exclusively from a single point at the center of the membrane.

The procedure is so simple that anyone with an interest in obtaining minimally disturbed synchronous cells should try it. There are, however, a few nuances that can be generalized. Of course it is essential to try the method first with a strain known to work effectively. For bacteria, it works best with *E. coli* B/r (ATCC 12407) and wild-type K12 (Helmstetter et al., 1992). It has also been shown to work very well with some other *E. coli* strains, *B. subtilis*, and *S. typhimurium* (Shehata and Marr, 1970; Holmes et al., 1980; Helmstetter et al., 1992). That said, any rod-shaped bacterium that does not aggregate or filament extensively during liquid culture is worth trying (after establishing adequate skill level with B/r). For cells other than *E. coli* B/r, the membrane filter might need to be pre-coated with an adhesive such as poly-D-lysine before use (Helmstetter et al., 1992). If a decision is made to perform long-term work with the technique, a specialized apparatus can be lathed from 15-cm diameter acrylic rods and cylinders as pictured in Thornton et al. (2002) and Helmstetter et al. (2003).

A video of the method, entitled “Cellular Baby Machine,” can be seen at (https://m.youtube.com/watch?v=hfGwetVg2gM). The video demonstrates the technique for mammalian cells using the specialized acrylic set-up, but it is performed in the same basic manner with bacteria and when using a Buchner funnel. In fact, the procedure is significantly easier for bacteria since sterilization of the apparatus is generally unnecessary.

## Fun with the cell cycle

Making babies can be fun, but it is not the only fun thing to try, as I hope to demonstrate in this final section. Visualizing the chromosome replication pattern during the division cycle of cells growing under a given set of conditions can sometimes seem so baffling that it is not worth the effort. However, understanding cellular responses to various treatments often demands that the replication pattern be taken into consideration. I suspect that the impression of complexity regarding this topic is related to the unfortunate abstruseness of some of the figures we and others have used to describe the cycle. The publication that contains the most complete description of the *I* + *C* + *D* model and its applications (Helmstetter et al., 1968) also contains, in retrospect, some impossibly complex figures. It is sometimes difficult and tiresome to look at a complex figure and plow through each aspect to get to the bottom line. Furthermore, the figure may not apply directly to the question at hand. Therefore, in this section I will describe the very simple method I use to determine replication patterns. This is exactly what I draw either on a lined pad for my own benefit or on a white board for students. In fact, the beginning of such a drawing appears on the chalkboard in Figure 2.

**Figure 5:**
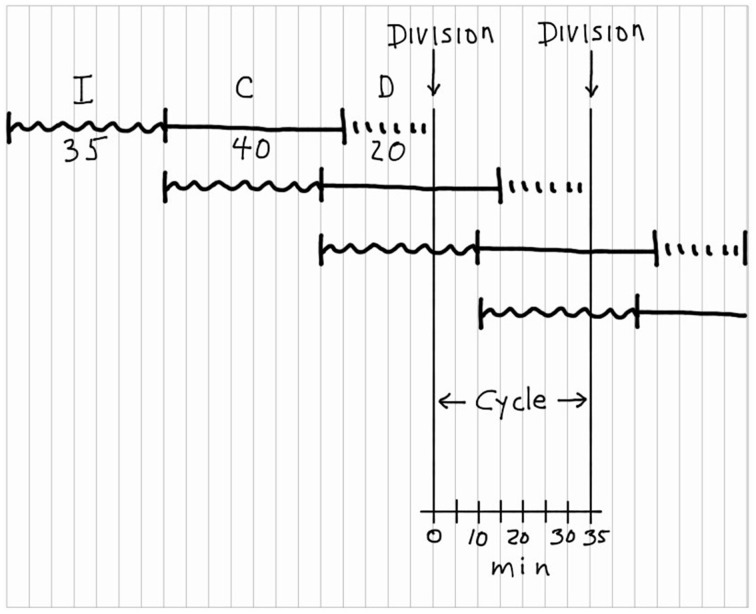
**Construction of a chromosomal replication pattern during the bacterial division cycle**. The construction, starting at the top left, is based on the (*I* + *C* + *D*) rule with *I* = 35 min, *C* = 40 min, and *D* = 20 min.

We will determine the moderately complex relationship between chromosome replication and cell division for the average *E. coli* B/r cell in a population growing with a doubling time of 35 min at 37°C. At this growth rate, reasonable values for *C* and *D* would be 40 min and 20 min, respectively. Referring to Figure 5, start at the top left by imagining a hypothetical cell with no initiation potential and draw a squiggly horizontal line, 35 min in duration, to represent *I*. *I* must be 35 min under these conditions because, as will be seen at the end of this exercise, *I* determines the doubling time. At the end of the *I* period, replication initiates, so draw a 40-min horizontal line to represent *C*, followed by a 20-min interrupted line to represent *D*. Then draw a long vertical line to represent cell fission. Now start the second *I* + *C* + *D* sequence below the first. Since *I* is continuous by definition, begin the next squiggly line at the time the *I* period ends in the first sequence, and then draw *C* for another 40 min, *D* for 20 min and another vertical line. At this point I would normally shout at the students, saying something like: “DON'T THINK! Just draw *I* + *C* + *D*, *I* + *C* + *D*, over and over until you have gone past the second vertical division line. If you try to think you will surely mess up, especially at more rapid growth rates.” Now we are done, and the division cycle for a cell with the given characteristics is shown between the two vertical lines representing division. It is important to note that the duplication process of *E. coli* can be described as a simple overlapping series of *I* + *C* + *D* sequences. Furthermore, the time between divisions is determined exclusively by *I* and independent of the durations of *C* and *D*.

**Figure 6:**
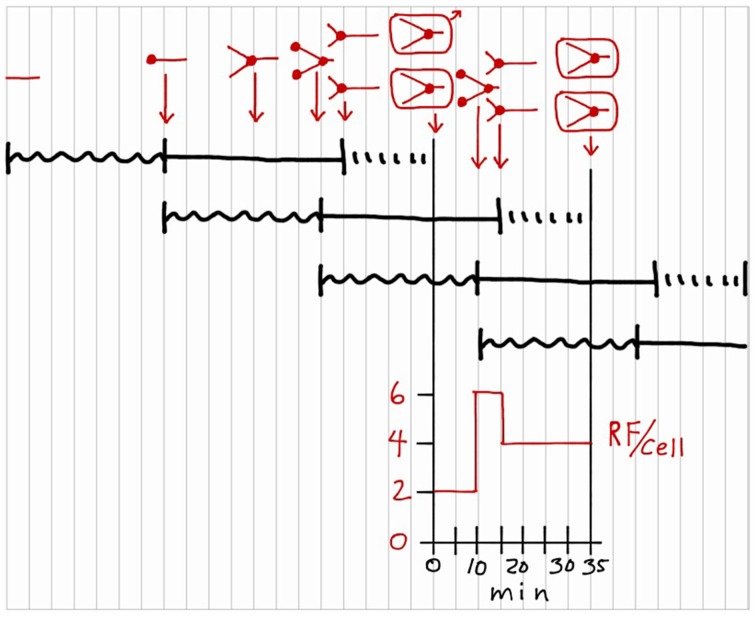
**Addition of chromosome configurations to a division cycle construction**. Chromosomal DNA is represented by red lines, with small filled circles indicating replication sites. The configurations in rectangles indicate cell divisions. Only one cell is followed after the first division. Number of replication forks per cell (RF/cell) is shown during the division cycle.

Thinking is now permitted to complete the exercise because the correctly drawn cycle is in front of us. The first step is to add chromosome configurations during the cycle to the sequences drawn in Figure 5. This process is presented in red in Figure 6, above the *I* + *C* + *D* sequences. Chromosomes are represented by straight lines rather than circles because circles are too hard to draw and unnecessary in this schematic representation. Again start at the beginning of the first sequence at the upper left by drawing a short horizontal line to represent a chromosome with no initiation potential residing in a hypothetical cell. The origin of replication is at the left end of the line, and the terminus at the right. Now, progress to the right, looking vertically as you go, to observe where the cell is located in each of the overlapping *I* + *C* + *D* sequences. At the end of the first *I* period, the chromosome initiates replication, as indicated by a small filled circle at the left end, and preparation begins for the next initiation event as shown in the second *I* + *C* + *D* sequence. After 20 min of the first *C* period, the chromosome is half replicated. After 35 min of the first *C* period, the chromosome is 35/40 replicated and new rounds of replication have initiated due to the second sequence, as indicated by two filled circles at the left end. Thus, the cell has begun to progress through two *C* periods simultaneously. Replication along the first sequence ends 5 min later so the cell now contains two chromosomes each 5/40 replicated. Twenty minutes later the cell divides due to the first sequence. Each daughter cell contains one chromosome that is 25/40 replicated due to the second sequence, and is also 25/35 of the way along preparation for the next initiation event due to the third sequence. This then is the chromosome configuration in a newborn cell in a culture growing with the given parameters. Chromosome replication during the division cycle of these cells is given by the chromosome configurations between the two vertical division lines. Configurations are shown for three key time points in the cycle, again determined by observing what is happening, vertically, in each *I* + *C* + *D* sequence. Note that cells growing at this rate are progressing along three reproductive paths simultaneously, such that the processes leading to a specific cell division began two division cycles in advance.

With the chromosome configurations completed, two additional aspects of the cycle can be determined. The graph beneath the division cycle in Figure 6 shows replication forks per cell (RF/cell) during the cycle, determined by counting the number of forks in the chromosome drawings and multiplying by two to account for bidirectional replication on the circular chromosome. Assuming the rate of DNA polymerization is constant during *C*, this graph shows the rate of chromosomal DNA synthesis during the cycle. For the first 10 min of the cycle, there are 2 RF/cell due to the single round of bidirectional replication on the chromosome. Then the RF/cell increases three-fold due to initiation of new rounds of bidirectional replication at the two origins on the replicating chromosome. Five minutes later, the intial round of replication finishes, and the cell is left with two chromosomes replicating bidirectionally for the last 15 min of the cycle, i.e., 4 RF/cell.

**Figure 7:**
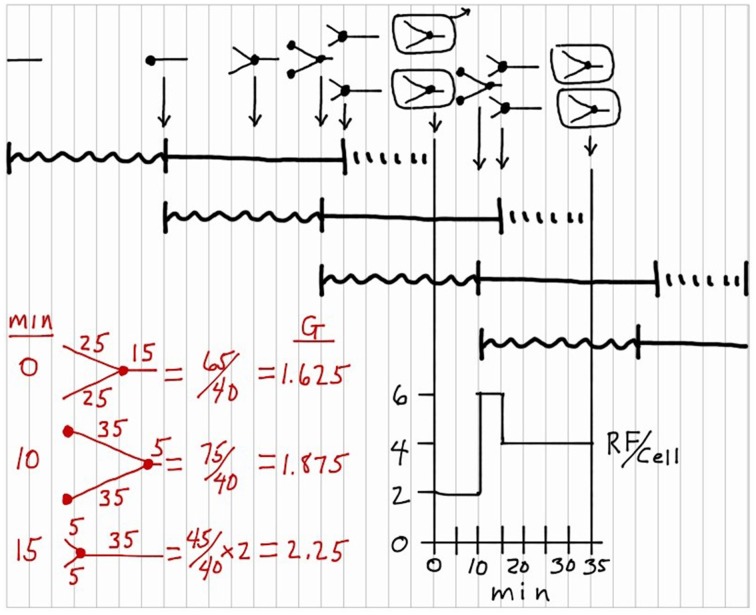
**Genome equivalents of DNA per cell (*G*) during the division cycle**. Calculations of the values for *G* are shown in red for three select times in the division cycle: 0, 10, and 15 min.

Finally, it is sometimes of interest to determine chromosomal DNA content per cell at various times in the cycle in terms of genome equivalents (*G*) (Cooper and Helmstetter, 1968). This calculation is shown in red in Figure 7 for three select times in the division cycle (0, 10, and 15 min). Again based on the idea that the replication rate is constant during *C*, this calculation is most easily visualized by recording the extent of chromosomal replication based on time rather than distance, and then dividing the sum of the times by *C* (40 min in this case) as shown. The average chromosomal DNA content in an exponentially growing culture can be determined with the equation: $G=\tau /Cln2\left[2^{(C+D)/\tau }-2^{D/\tau }\right]$, where τ equals doubling time.

The preceding shows an example of a simple method to sketch the division cycle of cells growing at any rate. Slower growing cells are easier to draw due to less overlap of *I* + *C* + *D* sequences, and more rapidly growing cells are more complex due to increased overlap but are still easy to do if you follow the rule of just drawing the *I* + *C* + *D* sequences without overthinking the issue. Simply change the values for *I*, *C*, and *D*, and follow the instructions in the preceding paragraphs. Should a difficulty be encountered, additional examples of this procedure can be found in Helmstetter (1996). It can be entertaining to consider cells with odd values for *I*, *C*, and *D* and then determine how they must behave.

A few additional points need to be made. The idea of overlapping *I* + *C* + *D* sequences for duplication appears to apply to many bacteria that divide by binary fission, but the durations of each step can vary considerably with temperature and culture conditions (e.g., Helmstetter et al., 1992, and references therein). For many *E. coli* strains, the values for *C* and *D* are roughly 40 and 20 min growing with doubling times between about 20 and 80 min at 37°C. Under these circumstances it is only necessary to measure τ to determine the chromosome replication pattern since τ equals *I*, and *C* and *D* are constants. On the other hand, in some strains *C* and *D* can be quite different, usually longer. During slow growth, one and sometimes two gaps in DNA synthesis appear in the cycle. When τ is between *C* and *C* + *D* min in duration, a gap exists at the end of the cycle during part or all of *D*. When τ is longer than *C* + *D* min, a gap also exists at the start of the cycle, designated the *B* period. But again, all of this can be seen by simply drawing the sequences as shown here with the appropriate values for the parameters. This analysis disregards dispersions in the values for the parameters in individual cells in a culture, but the purpose of the preceding was merely to display what happens in a single, average cell without regard to population variability.

My hope is that anyone who would like to run some experiments on synchronously dividing bacteria will try the procedure described here and see how simple yet useful the baby machine can be. I also hope the handy method for visualizing chromosome replication during the cell cycle will be found useful.

### Conflict of interest statement

The authors declare that the research was conducted in the absence of any commercial or financial relationships that could be construed as a potential conflict of interest.

## Footnotes

^1^The K-12 strains commonly employed at the time were generally motile, filament-forming and weakly adherent to the surfaces used for cell attachment, all of which would have caused experiments to fail or perform poorly. Once the technology was developed with the nonmotile, non-filamentous, strongly-adhesive strain B/r, the modifications necessary for use with K-12 became possible (Helmstetter et al., 1992).

^2^It was eventually assumed to be a culture media preparation problem, likely associated with the production of preconditioned media, which was not discovered until our laboratory moved to a new location 2 years later and the problem miraculously disappeared. Use of preconditioned medium was determined to be unnecessary, irreproducible and thus not recommended.
